# Prevalence and Correlates of Mental Health Symptoms and Well-Being Among Elite Sport Coaches and High-Performance Support Staff

**DOI:** 10.1186/s40798-022-00479-y

**Published:** 2022-07-06

**Authors:** Vita Pilkington, Simon M. Rice, Courtney C. Walton, Kate Gwyther, Lisa Olive, Matt Butterworth, Matti Clements, Gemma Cross, Rosemary Purcell

**Affiliations:** 1 The Centre for Youth Mental Health, The University of Melbourne, Melbourne, VIC Australia; 2 Elite Sports and Mental Health, Orygen, 35 Poplar Road, Parkville, VIC 3052 Australia; 3 School of Psychology, Faculty of Health, Deakin University, Geelong, VIC 3220 Australia; 4 Australian Institute of Sport, People Wellbeing and Engagement, Bruce, Australia

**Keywords:** Mental health, Well-being, Coach, Support staff, Sport, High performance

## Abstract

**Background:**

There is growing understanding of mental health needs in elite athletes, but less is known about the mental health of coaches and support staff who work within elite sport settings. This study examined the prevalence and correlates of mental health symptoms in elite-level coaches and high-performance support staff (HPSS) and compared rates against published elite athlete samples. A cross-sectional, anonymous, online survey was administered to coaches and HPSS working in Australia’s high-performance sports system. Main outcomes were scores on validated measures of psychological distress, probable ‘caseness’ for a diagnosable psychological condition, alcohol consumption and sleep disturbance.

**Results:**

Data were provided by 78 coaches (mean age = 46.4 years, 23.8% female) and 174 HPSS (mean age = 40.0 years, 56.7% female). Overall, 41.2% of the sample met probable caseness criteria, 13.9% reported high to very high psychological distress, 41.8% reported potential risky alcohol consumption and 17.7% reported moderate to severe sleep disturbance, with no statistically significant differences between coaches and HPSS. The most robust correlates of psychological distress and probable caseness were dissatisfaction with social support and dissatisfaction with life balance, while poor life balance was also associated with increased alcohol consumption and poor social support with sleep disturbance. Coaches and HPSS reported similar prevalence of mental health outcomes compared to rates previously observed in elite athletes, with the exception of higher reporting of alcohol consumption among coaches and HPSS.

**Conclusions:**

Elite-level coaches and HPSS reported levels of psychological distress and probable caseness similar to those previously reported among elite-level athletes, suggesting that these groups are also susceptible to the pressures of high-performance sporting environments. Screening for mental health symptoms in elite sport should be extended from athletes to all key stakeholders in the daily training environment, as should access to programs to support mental health and well-being.

**Supplementary Information:**

The online version contains supplementary material available at 10.1186/s40798-022-00479-y.

## Key Points

- Coaches and HPSS reported similar rates of mental health symptoms as previous elite athlete samples, suggesting that these groups are also susceptible to mental health concerns and pressures within high-performance sports settings.
- Satisfaction with life balance and satisfaction with social support appear to act as key protective factors for mental health. Efforts aiming to enhance these psychosocial factors have the potential to directly enhance coach and HPSS well-being.
- Unlike previous research with elite athlete samples, no significant gender differences in mental health symptomatology were observed, suggesting a differential role of gender between athletes and members of the daily training environment in terms of mental health and well-being outcomes.

## Background

There is increasing awareness of the rates and nature of mental health symptoms experienced among elite athletes [1], with approximately 1 in 3 currently competing athletes reporting symptoms of common mental health concerns, such as anxiety, depression and psychological distress [2, 3]. These rates are consistent with or higher than those observed in general community samples [2, 3]. A number of sport-specific risk factors for elite athlete mental ill health have been identified to date, including performance pressures, serious (including chronic) injury, maladaptive perfectionism, maladaptive coping strategies and inadequate social support [1, 3, 4]. There is comparatively limited understanding, however, of the mental health and well-being of coaches and high-performance support staff (HPSS) who work alongside athletes within elite sport settings [5]. The latter group includes a wide network of professionals who support athlete health, functioning and performance, such as athletic trainers, physiotherapists, nutritionists, strength and conditioning coaches, sports psychologists, and athlete well-being and engagement managers.

Coaches and HPSS operate within a broader ‘ecology’ of elite sporting environments [6]. As argued in recent sports mental health position papers and frameworks, in addition to key responsibilities related to athlete performance, athlete health, team cohesion and organizational functioning, coaches and HPSS also have critical roles in promoting and protecting the well-being of athletes [1, 7, 8], including promoting the importance of mental health, recognizing experiences of mental ill health as legitimate, identifying emerging symptoms of mental ill health and encouraging appropriate help-seeking. Coaches and HPSS may share characteristics already established as risk factors for mental ill health in elite athletes (e.g., pressure to perform, maladaptive perfectionism) and may experience specific role-related stressors that place them at risk for mental ill health, such as insecure employment, unclear roles, feeling undervalued and doubting their coaching (or role) competencies [9]. Understanding coach and HPSS mental health and well-being is therefore pivotal for understanding the mental health needs and resourcing requirements within elite sports settings holistically and has additional potential benefits that may flow from promoting athlete well-being and creating a flourishing elite sports environment. However, it is currently unclear whether elite-level coaches and HPSS experience mental health symptoms at a similar rate to elite athletes, or whether known risk factors for mental ill health in athletes (such as female gender [10] and individual as opposed to team sport participation [11]) also confer risks to coach and HPSS mental health.

A study by Smith and colleagues [12] highlighted relatively high mental health need among coaches, with 55% reporting that they had experienced mental illness. However, this sample comprised coaches from a range of professional backgrounds and performance levels (including *n* = 35 elite coaches) and most study findings were not reported separately for the elite coach group. To date, only three studies have examined the rates of common mental health symptoms among elite coaches, with estimates ranging from 14% for depressive symptoms in a sample of 69 New Zealand coaches [13], to 39.5% for mixed depression/anxiety symptoms among 119 Dutch and Flemish coaches [14]. A recent study by Åkesdotter and colleagues [15] investigated a small sample of high-performance coaches (*n* = 34) seeking treatment from a psychiatric outpatient service (in addition to 221 elite athletes seeking support from the same service). Nearly all the coaches presenting to the outpatient service (93%) presented with anxiety disorders, while 28% presented with major depressive disorder. The authors also reported high prevalence (72%) of stress-related disorders among coaches (compared to 25% among the athlete sample).

Coaches that are contemplating or recently experiencing retirement report even higher rates of mental health symptomatology [13, 16], which is consistent with a pattern observed in retired or former elite athletes [2]. Known risk factors for mental ill health among coaches include lack of life balance, burnout, performance-based stressors (e.g., lack of athlete commitment, poor performance and poor performance preparation), organizational stressors (e.g., poor organizational communication, unclear roles, conflict) and personal challenges (e.g., missing children’s education, long periods away from home) [13, 14, 17]. Of note, organizational but not performance stressors have been found to be predictive of increased depression/anxiety symptoms [14]. In the limited available evidence, coaches have identified job security, professional and personal growth opportunities, high autonomy support and life balance as protective mental health factors [18, 19].

To date, no research has systematically examined rates of mental health concerns among HPSS, although Hill and colleague’s [5] qualitative study of members of the daily training environment (inclusive of coaches and HPSS) reported key perceived protective factors for mental health to include social support, leadership and organizational culture. Key perceived risk factors included high workload, competitive performance and isolation [1]. However, risk and protective factors for mental ill health in HPSS are yet to be studied using quantitative methods.

This study examined the rates and correlates of mental health symptoms in a large cohort of elite-level coaches and HPSS. Associated aims were to (a) compare rates of symptoms to published elite athlete samples to determine whether mental health profiles differ between these groups; (b) explore similarities and differences between coach vs HPSS profiles (in relation to symptoms of mental ill health, adverse events, social support, life balance and strategies for managing stress and mental well-being); and (c) explore correlates of mental health in this group (such as satisfaction with life balance and social support). It was hypothesized that [H1] symptom rates of common mental health concerns in coaches and HPSS would be similar to those reported by elite athletes; [H2] coaches and HPSS would have similar profiles in terms of mental health symptoms; and [H3] putative risk factors would be associated with symptoms of common mental health concerns, including sport-related factors (e.g., frequency of sport-related travel, missing significant life events due to sport-related travel) and general risk factors (e.g., number of lifetime and past year adverse events, previous mental health treatment, female gender), while known protective factors (e.g., life balance and social support) would be negatively associated with symptoms of common mental health concerns.

## Methods

### Participants

All coaches and HPSS employed by national sporting organizations (NSOs) and national institute networks (NINs) in Australia’s high-performance sport system (the Australian Institute of Sport: AIS) were invited to participate in an anonymous online survey regarding their mental health and well-being. HPSS included high-performance directors, physiotherapists, nutritionists, medical doctors, sports psychologists, strength and conditioning coaches, athlete well-being and engagement advisors, and others involved in the daily training environment. No exclusion criteria were applied for survey participation other than the ability to read English.

### Procedure

A link to the online survey was provided to potential participants via text message or email (depending on each participant’s preferred AIS registered contact details). The survey was built by Orygen’s research database management team and hosted on a secure research management platform. The survey was open between March 16 and May 31, 2020. Participants completed the survey at a place and time of their choosing. The survey took approximately 20 min to complete and was enabled for completion on any electronic device (i.e., smartphone, computer or tablet).

All participants were provided with information about the purpose and nature of the survey prior to commencing, and informed consent was implied by participants choosing to click to ‘enter’ the survey. The survey concluded with participants being directed to a debriefing statement that included contact details for relevant mental health support services and the project investigators, should the participant wish to discuss their experience with the survey or any concerns regarding their responses. The research was approved by, and conducted in accordance with, the ethical standards of the University of Melbourne Human Ethics Research Committee (#13718) and the 1964 Helsinki Declaration.

### Survey Content

The survey was developed in consultation with AIS staff and Paralympics Australia based on a previous project [3]. Wherever possible, validated scales that were developed for, or used with, elite athletes and shown to be reliable in prior research were used in the survey, in order to enable comparisons.

#### Background Information/Demographics

Basic demographic details were collected, including participant age, gender, relationship status, sexual orientation and highest level of education. Participants were also asked about employment-related characteristics, including the number of years they had worked in high-performance sport, and their current employment status with a national sporting organization or institute and whether they had engaged in any voluntary or paid employment in the past month in addition to their sporting role. Sport-related characteristics included the type of sport(s) they coached or supported (e.g., individual or team-based), whether they were currently preparing athletes for upcoming competition, frequency of sport-related travel over the past 12 months, whether they had missed significant life events due to sport-related travel and concerns for safety while traveling for their sport in the 12 months prior to the survey. Participants were also asked if they had previously accessed treatment for a psychological issue or mental health problem.

#### Symptom Outcome Measures

*Mental Health Symptoms and Probable Caseness* The 28-item General Health Questionnaire (GHQ-28; [20]) was used to assess mental health symptoms in the past 4 weeks. The GHQ-28 includes a total score and four subscale scores, which assess somatic complaints (e.g., ‘*been feeling run down and out of sorts*’), anxiety and insomnia (e.g., ‘*lost much sleep over worry*’), social dysfunction (e.g., ‘*felt on the whole you were doing things well*’) and severe depression (e.g., ‘*felt that life is entirely hopeless*’). Items are scored *0* = *not at all, 1* = *no more than usual, 2* = *more than usual and 3* = *much more than usual*, with GHQ-28 total scores ranging from 0 to 84.

The GHQ-28 can also be scored as a categorical measure, which can be used to indicate the proportion of participants meeting the threshold for ‘probable caseness’ (the reporting of psychological symptoms at a level that would usually warrant treatment from a health professional). To calculate caseness, binary coding is applied to the four response items (*0* = *not at all* or *no more than usual*, *1* = *more than usual* or *much more than usual*) and the total score with this binary coding is calculated (range = 0–28). The cutoff score used was 5 or more indicating probable caseness, as per [21] and to allow comparisons with a comparable athlete sample [3].

*Psychological Distress* The Kessler-10 (K-10; [22]) was used to measure psychological distress. The K-10 requires respondents to rate the frequency at which they have experienced symptoms of psychological distress over the previous 4 weeks (e.g., ‘*about how often did you feel that everything was an effort*’). Items are scored *1* = *none of the time, 2* = *a little of the time, 3* = *some of the time, 4* = *most of the time, 5* = *all of the time*, where K-10 total scores range 10–50.

*Risky Alcohol Consumption* The Alcohol Use Disorder Identification Tool-Concise (AUDIT-C; [23]) was used as a brief 3-item measure of alcohol consumption that identified individuals at risk of risky alcohol consumption. Items enquire about frequency and quantity of alcohol consumption, where each item is scored 0–4 and total scores range from 0 to 12. To allow for comparisons with the previously published literature (e.g., [15]), we used a cutoff score equal to or above 4 for women and 5 for men to determine potentially risky alcohol consumption (also see footnote in Table 2 for the rate meeting risky alcohol consumption using the more stringent IOC recommended cutoffs [24]).

*Sleep* The Athlete Sleep Screening Questionnaire (ASSQ; [25]) was used to assess possible sleep disturbance. This measure includes five items that enquire about satisfaction with recent sleep quality, sleep duration, sleep onset latency, sleep maintenance and use of sleep medication. ASSQ total scores (range = 0–17) can be categorized into levels of sleep disturbance (5–7 = mild disturbance, 8–10 = moderate disturbance, 11–17 = severe disturbance) [26].

#### Comparison Data

For each of the main mental health outcome measures, comparisons were made between coaches and HPSS versus published elite athlete data. Where possible, athlete data were obtained from a study with a comparable athlete sample, comprising athletes aged 17 years and older contracted with the AIS [3]. Where comparison data with this sample were unavailable (i.e., for the AUDIT-C and ASSQ), data from studies with elite athlete samples that used the same cutoff scores as described above were used [15, 27, 28].

#### Psychosocial Correlates

*Quality of Life* Past 4-week quality of life was assessed using a single item from the World Health Organisation [29] (‘*Thinking about your life in the last 4 weeks, how would you rate your quality of life?’*), rated on a five-point scale (*1* = *very poor, 2* = *poor, 3* = *neither good nor poor, 4* = *good, 5* = *very good*).

*Satisfaction with Life* Satisfaction with life was assessed using the Satisfaction with Life Scale [30], which includes 5 items (e.g., ‘*So far, I have gotten the important things I want in life*’) rated on a 7-point scale (*1* = *strongly disagree, 2* = *disagree, 3* = *slightly disagree, 4* = *neither agree or disagree, 5* = *slightly agree, 6* = *agree, 7* = *strongly agree*). Total scores range from 5–35.

*Life balance* was assessed using a single Y/N item (*‘Are you satisfied with your life balance, e.g., managing your sport, work, social life, family, sleep, *etc*.?’*).

*Social support* was assessed using six questions, which enquired about presence (Y/N) of support, main source of support, level of satisfaction with support (*rated 1* = *totally dissatisfied to 7* = *completely satisfied*), and experiences of social isolation (feeling of lacking companionship, feeling left out, feeling isolated; rated in terms of frequency *1* = *hardly ever, 2* = *some of the time, 3* = *often*). The social support items were assessed individually, rather than summed.

*Adverse life events* were assessed over the past year and lifetime. Thirteen items were included (see Additional file 1: Table S1), which included experiences of general adverse events (e.g., *‘A person close to me died’*) and sport-specific events (e.g., ‘*I felt under-valued or under-paid’; ‘I was harassed or abused on social media’*), each rated *0* = *no, never, 1* = *yes, 2* = *yes, past year*.

*Strategies for managing mental well-being* was assessed by providing participants with a list of strategies commonly used to manage stress or mental well-being (e.g., ‘*using relaxation techniques’; ‘talking with a friend/partner’*), with participants indicating which of the strategies they used in their daily life with binary Y/N responses (see Additional file 1: Table S2).

*Concern about COVID-19* At the time of the planned survey implementation, the COVID-19 pandemic was emerging, with attendant restrictions. A question was included in the survey to assess COVID-19 concern (Y/N), with affirmative responses asked to specify their level of concern about the pandemic (*1* = *a little concerned, 2* = *somewhat concerned, 3* = *greatly concerned*) and specific concerns related to the pandemic via providing a list of potential concerns, as well as an open-ended response option for other concerns.

### Data Analyses

Categorical variables were summarized using frequencies and percentages, and continuous variables were summarized using mean and standard deviation. Group comparisons were made to examine possible differences according to demographic characteristics (e.g., gender, role) and for comparing coach and HPSS data to published data with elite athlete samples. Group comparisons for continuous outcome measures were made using independent samples *t* tests (measure of effect size = Cohen’s *d*), while comparisons for categorical outcome measures were made using chi-square (measure of effect size = Cramer’s *V*). All outcomes have been evaluated as statistically significant at the *p* ≤ 0.01 level, to reduce the risk of Type I error.

Separate regression models were developed for each major outcome: caseness (according to GHQ-28), psychological distress (K-10), alcohol consumption (AUDIT-C) and sleep (ASSQ). For caseness, which had a dichotomous outcome (i.e., meets caseness criteria vs does not meet criteria), logistic regression analysis was used. Continuous outcomes (K-10, AUDIT-C and ASSQ) were assessed for significant departures from normality using the Shapiro–Wilk test, and quantile median regression was used (where median scores were used instead of mean scores to account for departures from normality).

A two-stage analysis was performed for each model, where unadjusted associations were examined between the outcome measure and possible correlates, which included demographic variables (e.g., gender, age), sport-related characteristics (e.g., individual/team sport, frequency of sport-related travel), employment-related variables (e.g., number of years working in high-performance sport), number of adverse life events (past year and lifetime) and other possible psychosocial correlates (e.g., previous or current mental health treatment, satisfaction with life balance, satisfaction with social support). Additionally, the presence of concern about the COVID-19 pandemic (Y/N) and date of survey completion (pre- or post-announcement about the postponement of the Tokyo Olympics and Paralympics) were included as possible correlates in each regression model. In the second stage (following the identification of significant correlates), only significant correlates were entered into the adjusted model, therefore controlling for the effects of each salient variable from the unadjusted model. All analyses were conducted using IBM SPSS Statistics 25 and R Version 4.0.0.

## Results

### Descriptive Statistics

A total of 78 coaches and 174 HPSS completed some or all of the survey (i.e., at least two mental health outcome measures), representing a valid response rate of 31.5%.

Participants reported working across 32 sports, with the majority involved in individual sports (55.0% of coaches; 66.7% of HPSS), rather than team-based sports. The majority of participants were born in Australia (77.8% of coaches; 83.9% of HPSS), married (72.5% of coaches; 56.4% of HPSS), identified as heterosexual (95.1% of coaches; 96.1% of HPSS), and had completed a university degree (67.5% of coaches; 87.8% of HPSS). The mean age was 46.4 years ( = 8.78) for coaches and 40.0 years (SD = 9.65) for HPSS. Among coaches, more individuals were male-identifying (76.3%) than female-identifying (23.8%), whereas there was a more even gender distribution among HPSS (56.7% female-identifying and 41.1% male-identifying). A small number (*n* < 5) preferred not to disclose their gender. Approximately two-thirds were employed by a NSO (66.2% of coaches; 64.2% of HPSS), while approximately one-third were employed by a NIN (33.8% of coaches; 35.8% of HPSS). The majority reported they had worked in high-performance sport for 10 or more years (60.5% of coaches; 51.1% of HPSS). Participant demographic and sport-related characteristics are summarized in Table 1.

**Table 1:** Demographics and sport-related characteristics

Demographic and sport-related characteristics	Coaches % (*n*)	HPSS % (*n*)
Mean age (SD)	46.4 (8.78)	40.0 (9.65)
Gender (% female)	23.8 (19)	56.7 (102)
Australian-born	77.8 (63)	83.9 (151)
*Employer*		
National Sporting Organization	66.2 (49)	64.2 (111)
National Institute Network	33.8 (25)	35.8 (62)
*Employment modality*		
Full time	83.8 (67)	67.2 (121)
Part time (> 0.5 FTE)	6.3 (5)	14.4 (26)
Part time (< 0.5 FTE)	3.8 (–)	7.8 (14)
Casual	6.3 (5)	10.6 (19)
*Other work in previous month*		
Voluntary	18.5 (15)	8.9 (16)
Paid casual	11.1 (9)	12.8 (23)
Paid part time	11.1 (9)	15.0 (27)
Paid full time	4.9 (–)	7.8 (14)
*Duration working in high-performance sport*		
Less than 12 months	0.0 (–)	2.2 (–)
1 year	2.5 (–)	3.9 (7)
2–3 years	6.2 (5)	14.4 (26)
4–5 years	11.1 (9)	11.1 (20)
6–9 years	19.8 (16)	17.2 (31)
10 years or more	60.5 (49)	51.1 (92)
*Sport type*		
Individual sport	55.0 (44)	66.7 (112)
Team sport	45.0 (36)	33.3 (56)
Preparing athletes for upcoming competition	66.3 (53)	76.0 (136)
*Time travelling for sport (past 12 months)*		
Less than 1 month	14.8 (12)	46.4 (83)
1–2 months	39.5 (32)	29.6 (53)
3–4 months	29.6 (24)	19.6 (35)
5–6 months	7.4 (6)	2.2 (–)
6 months or more	8.6 (7)	2.2 (–)
Missed significant life events due to sport-related travel	71.6 (58)	49.7 (89)
*Concerned for safety while travelling*		
Personal concern	16.0 (13)	6.1 (11)
Family/friends expressed concern	28.4 (23)	21.2 (38)

Dash (–) indicates *n* < 5

### Psychosocial Variables: Help-Seeking, Adverse Events, Social Support, Quality of Life and Life Balance

Approximately 1 in 3 participants reported they had sought treatment for a psychological issue or mental health problem at some stage (34.6% of coaches, 34.4% of HPSS). The reporting of adverse life events was similar between coaches and HPSS (see Additional file 1: Table S1). The most commonly reported lifetime adverse events were the death of a close person (54.3% of coaches; 49.2% of HPSS), a relative or close friend suffering from serious illness, injury or assault (reported by 51.9% of coaches and 49.7% of HPSS), and feeling undervalued or underpaid (54.3% of coaches; 48.6% of HPSS). This is consistent with adverse events reported by elite athletes [10].

Almost all participants reported having social support (92.1% of coaches; 92.8% of HPSS), and the majority reported satisfaction with their social support (75.7% of coaches; 85.3% of HPSS). The majority of coaches (85.5%) and HPSS (87.9%) reported that their main source of support was someone outside their sport, most commonly a spouse/partner, followed by a friend, then a parent. Only 5.3% of coaches and 1.2% of HPSS reported their main source of support was someone within their sport (such as a member of HPSS or sport psychologist), while few reported mainly receiving support from a mental health professional (no coaches, 1.7% of HPSS).

Satisfaction with life balance was endorsed by less than half of the coach sample (43.6%) and just over half of the HPSS sample (54.9%). The majority of participants rated their quality of life as ‘good’ or ‘very good’ (64.9% of coaches; 73.8% of HPSS), while a small number rated their quality of life as ‘poor’ or ‘very poor’ (9.1% of coaches; 8.7% of HPSS).

### Strategies for Managing Stress and Mental Well-Being

Participants endorsed using a range of strategies to manage their stress and mental well-being, with few differences observed between coaches and HPSS. The most commonly utilized strategies included talking with a friend/partner (reported by 71.4% of coaches and 83.8% of HPSS), exercising for pleasure (67.5% coaches; 80.2% HPSS), engaging in routine enjoyable activities such as walking the dog or listening to music (59.7% coaches; 74.3% HPSS) and sleeping (61.0% coaches; 71.9% HPSS). A small number reported doing nothing to manage their stress or mental well-being (6.5% coaches; 3.6% HPSS), and even fewer reported they did not know how to manage their stress or mental well-being (3.9% coaches; 1.8% HPSS).

### Possible Impacts of COVID-19

Given that data collection occurred during the onset of the COVID-19 pandemic (March–May 2020), we examined possible impacts of concern about COVID-19 on the major outcome measures (GHQ-28, K-10, ASSQ and AUDIT-C). Independent samples t tests were used to examine concern about the presence of COVID-19 (Y/N) and outcome measures, with results demonstrating no significant differences between participants who reported feeling concerned about the pandemic compared to those who did not (in all instances, *p* > 0.01 for all).

### Comparisons According to Role, Gender and Sport Type

The rates of mental health symptoms for coaches and HPSS are presented in Table 2. While a significant proportion met the threshold for caseness (~ 40%) and risky alcohol consumption (41.8%), the reported rates of high to very high psychological distress (13.9%) were comparatively lower. For all major outcome measures (i.e., the K-10, GHQ-28, AUDIT-C and ASSQ), group comparisons were made according to role (coach vs HPSS), gender (male vs female) and sport type (individual vs team sport). No significant differences were observed according to role (*p* > 0.01 for all). Within the coach sample, no significant group differences according to gender or sport type were observed (all *p* > 0.01). In contrast, male HPSS reported significantly higher alcohol consumption than females on the basis of AUDIT-C total scores (mean = 3.77 vs 2.77, *p* = 0.004; medium effect: Cohen’s *d* = 0.47). Additionally, a larger proportion of female HPSS reported that they had sought mental health treatment at some stage relative to their male counterparts (43.1% vs 23.0%, *p* = 0.006), although this reflected a small effect (Cramer’s *V* = 0.21).

**Table 2:** Reported mental health symptoms among coaches and HPSS compared to published elite athlete samples

Measure		Coaches	HPSS	Published athlete samples
*GHQ-28*				
Total score	M (SD)	20.62 (9.87)	20.70 (9.95)	19.66 (10.81) [3]
Somatic complaints	M (SD)	5.56 (3.42)	5.68 (3.76)	5.53 (3.58) [3]
Anxiety/insomnia	M (SD)	6.27 (4.53)	6.38 (4.46)*	5.47 (4.51) [3]*
Social dysfunction	M (SD)	7.58 (2.39)	7.82 (2.71)*	7.17 (2.64) [3]*
Severe depression	M (SD)	1.27 (2.58)	0.77 (1.53)**	1.50 (2.77) [3]**
Caseness	%	43.6	40.1	35 [3]
*K-10*				
Total score	M (SD)	15.58 (4.95)	15.75 (5.25)	16.40 (5.89) [3]
High to very high distress	%	10.3	15.5	17.7 [3]
*ASSQ*				
Total score	M (SD)	5.73 (2.48)	5.09 (2.35)	5.3 (–) [41]
No sleep disturbance	%	32.5	40.7	43 [27]
Mild sleep disturbance	%	44.2	44.2	41 [27]
Moderate sleep disturbance	%	18.2	12.8	16 [27]
Severe sleep disturbance	%	5.2	2.3	0 [27]
*AUDIT-C*				
Total score	M (SD)	3.88 (2.55)	3.18 (2.12)	4.28 (2.61) [28]
Risky alcohol consumption	%	48.1**	39.0**	25.8 [15]**
Satisfaction with life total score	M (SD)	25.22 (5.75)	25.91 (5.40)	26.6 (5.95) [3]

No statistically significant differences were found between coaches and HPSS. Comparisons were made between the coach sample and published athlete samples and between the HPSS sample and published athlete samples, *p < .01, **p < .001. For GHQ-28 caseness, the cutoff score 5 or more has been used. For risky alcohol consumption, the cutoff score 4 or more has been used for females and 5 or more has been used for males. Using cutoffs recommended by the IOC [24] of 3 or more for women and 4 or more for men, 56.6% of participants met risky drinking criteria (59.7% of coaches, 55.2% of HPSS). K-10 scores between 22 and 50 were suggestive of ‘high to very high’ distress

### Comparisons Between Coach and HPSS Outcomes and Published Elite Athlete Data

Separate comparisons were made between the coach and HPSS samples and published elite athlete data (see Table 2; also see community comparisons in Additional file 1: Table S3). The only statistically significant difference observed in relation to coaches was a higher reporting of risky/hazardous alcohol consumption (*X*^2^ (1, *N* = 450) = 13.60, *p* < 0.001; small effect: Cramer’s *V* = 0.17). Overall, 48.1% of coaches met hazardous/risky drinking criteria.

In comparison with published athlete data, HPSS scored significantly higher (i.e., worse) on two of the GHQ-28 subscales: the anxiety/insomnia subscale (*p* = 0.008; Cohen’s *d* = 0.20) and the social dysfunction subscale (*p* = 0.002; Cohen’s *d* = 0.24). However, HPSS reported significantly lower (i.e., better) scores on the severe depression subscale than the comparable athlete sample (*p* < 0.001; Cohen’s *d* = 0.33). Finally, HPSS reported significantly lower scores than athletes on the ASSQ (*p* < 0.001; Cohen’s *d* = 0.28), suggesting less sleep disturbance among HPSS compared to the athlete sample.

As per coaches, HPSS were more likely than athletes to report risky alcohol consumption, *X*^2^ (1, *N* = 545) = 7.61, *p* < 0.01 (weak effect: Cramer’s *V* = 0.12). Overall, 39.0% of HPSS met risky drinking criteria. No differences were observed between HPSS and a comparable athlete sample on the K-10 total score or the proportion reporting ‘high to very high’ distress (*p* > 0.01 for both).

### Correlates of Mental Health and Alcohol Consumption

Given the overall lack of differences between coach and HPSS mental health outcome measures, data were pooled to examine correlates of mental health and well-being symptoms, using coaches and HPSS as a combined group. Relationships were assessed between each outcome and a range of possible correlates. Tables 3, 4 and 5 display the correlates that were significant in the univariable and/or multivariable modeling stage.

**Table 3:** Unadjusted and adjusted odds ratios for GHQ-28 caseness; multi-predictor logistic regression for GHQ-28 caseness

Factor	Level	Unadjusted OR (95% CI) *p* value	Adjusted OR (95% CI) *p* value
Age (years)		**0.96 (0.93, 0.99) 0.004**	0.96 (0.92, 0.99) 0.041
Relationship status	Single/never married	Reference	Reference
	Partnered	0.44 (0.13, 1.47) 0.182	0.77 (0.17, 3.52) 0.738
	De facto/living together	0.46 (0.17, 1.23) 0.122	0.46 (0.13, 1.56) 0.211
	Married	0.66 (0.31, 1.42) 0.286	1.59 (0.55, 4.59) 0.388
	Separated	**1.00 (0.00, 0.00) < .001**	–
	Divorced	0.22 (0.04, 1.20) 0.081	0.22 (0.03, 1.70) 0.146
	Widowed	N/A	–
	Not reported	0.88 (0.05, 15.37) 0.932	–
Sexual orientation	Heterosexual	Reference	Reference
	Same sex attracted	**1.00 (0.00, 0.00) < .001**	–
	Bisexual	**1.00 (0.00, 0.00) < .001**	–
	Don't know	2.09 (0.34, 12.71) 0.426	2.81 (0.21, 37.32) 0.434
	Other	N/A	–
	Not reported	**1.00 (0.00, 0.00) < .001**	–
Gender	Binary female	1.22 (0.73, 2.02) 0.452	1.24 (0.63, 2.44) 0.530
	Binary male	Reference	Reference
	Non-binary	N/A	–
	Not sure	**1.00 (0.00, 0.00) < .001**	**–**
	Not reported	1.00 (0.00, 0.00) 0.000	–
Aboriginal or Torres Strait Islander	No	Reference	Reference
	Yes	**1.00 (0.00, 0.00) < .001**	–
	Not reported	0.57 (0.30, 1.10) 0.095	–
Highest educational level completed	No formal schooling	**1.00 (0.00, 0.00) < .001**	–
	Primary school	N/A	–
	Year 10–11	0.50 (0.05, 4.87) 0.549	–
	Year 12	1.24 (0.51, 3.02) 0.628	1.68 (0.59, 4.79) 0.331
	University degree	Reference	Reference
	Trade/Apprenticeship/TAFE	1.49 (0.59, 3.75) 0.393	2.51 (0.78, 8.05) 0.122
	Not reported	**1.00 (0.00, 0.00) < .001**	–
Accommodation	Living at family home	Reference	Reference
	Living with host family	N/A	–
	Living in college/university	N/A	–
	Renting	1.75 (0.52, 5.94) 0.366	2.08 (0.49, 8.88) 0.324
	Own home (mortgage)	1.04 (0.32, 3.33) 0.954	0.83 (0.21, 3.20) 0.788
	Own home (outright)	0.61 (0.15, 2.43) 0.483	0.98 (0.18, 5.37) 0.980
	Other	**1.00 (0.00, 0.00) < .001**	–
	Not reported	1.60 (0.08, 31.77) 0.758	–
Satisfaction with life balance	Yes	**0.25 (0.15, 0.43) < .001**	**0.40 (0.21, 0.77) 0.006**
	No	Reference	Reference
	Not reported	0.74 (0.05, 12.15) 0.835	–
Satisfaction with social support	Yes	**0.62 (0.49, 0.79) < .001**	**0.65 (0.49, 0.87) 0.004**
	No	Reference	Reference

Bold indicates the factor is significant at *p* < .01 or .001 level. Dash (–) indicates there were insufficient numbers in the group

**Table 4:** Unadjusted and adjusted odds ratios (beta-coefficients) for median total K-10 scores; multi-predictor median regression for psychological distress

Factor	Level	Unadjusted β-coefficient (95% CI) *p* value	Adjusted β-coefficient (95% CI) *p* value
Age (years)		**− 0.16 (− 0.23, − 0.09) < .001**	**− 0.09 (− 0.14, − 0.03) 0.003**
Sexual orientation	Heterosexual	Reference	Reference
	Same sex attracted	2.00 (**− **3.26, 7.26) 0.455	0.33 (**− **3.82, 4.47) 0.877
	Bisexual	2.00 (**− **8.46, 12.46) 0.707	2.85 (**− **5.50, 11.19) 0.502
	Don't know	**14.00 (9.68, 18.32) < .001**	1.46 (**− **2.26, 5.17) 0.440
	Other	–	–
	Not reported	**− **3.00 (**− **13.46, 7.46) 0.573	–
Any psychological or mental health treatment	Yes	**2.00 (0.73, 3.27) 0.002**	0.72 (**− **0.54, 1.97) 0.261
	No	Reference	Reference
	Not reported	–	–
Current psychological or mental health treatment	Yes	**5.00 (2.34, 7.66) < .001**	1.15 (**− **1.34, 3.65) 0.364
	No	Reference	Reference
	Not reported	–	–
Satisfaction with life balance	Yes	**− 3.00 (− 4.20, − 1.80) < .001**	**− 1.93 (− 3.25, − 0.61) 0.004**
	No	Reference	Reference
	Not reported	2.00 (**− **7.52, 11.52) 0.679	–
Satisfaction with social support	Yes	**− 1.75 (− 2.23, − 1.27) < .001**	**− 1.38 (− 1.91, − 0.86) < 0.001**
	No	Reference	Reference
Felt undervalued or underpaid (ever)	Yes	**2.00 (0.48, 3.52) .010**	0.556 (**− **0.57, 1.68) .332
	No	Reference	Reference

Bold indicates the factor is significant at *p* < .01 or .001 level. Dash (–) indicates there were insufficient numbers in the group

**Table 5:** Unadjusted and adjusted odds ratios (beta-coefficients) for median total ASSQ scores; multi-predictor median regression for sleep disturbance

Factor	Level	Unadjusted β-coefficient (95% CI) *p* value	Adjusted β-coefficient (95% CI) *p* value
Aboriginal or Torres Strait Islander	No	Reference	Reference
	Yes	**− **1.00 (**− **5.54, 3.54) 0.665	–
	Not reported	**− 1.00 (− 1.70, − 0.30) 0.005**	–
Highest educational level completed	No formal schooling	–	–
	Primary school	–	–
	Year 10–11	1.00 (**− **2.04, 4.04) 0.518	1.00 (**− **1.94, 3.94) 0.503
	Year 12	1.00 (**− **0.35, 2.35) 0.146	0.50 (-0.63, 1.63) 0.386
	University degree	Reference	Reference
	Trade/Apprenticeship/TAFE	**2.00 (0.59, 3.41) 0.006**	1.50 (0.26, 2.74) 0.018
	Not reported	**− **1.00 (**− **7.03, 5.03) 0.744	N/A
Satisfied with life balance	Yes	**− 1.00 (− 1.60, − 0.40) 0.001**	**− **1.50 (**− **2.20, **− **0.80) 0.018
	No	Reference	Reference
	Not reported	N/A	N/A
Satisfaction with social support	Yes	**− 0.75 (− 1.05, − 0.45) < .001**	**− 0.50 (− 0.79, − 0.21) 0.001**
	No	Reference	Reference

Bold indicates the factor is significant at *p* < .01 or .001 level. Dash (–) indicates there were insufficient numbers in the group

#### Correlates of Psychiatric Caseness/Morbidity (GHQ-28)

As Table 3 indicates, a range of factors were associated with meeting the threshold for caseness on the GHQ-28 in the unadjusted model. The variables that remained significant in the adjusted model were satisfaction with social support and satisfaction with life balance, both of which predicted *lower* odds of meeting caseness criteria.

#### Correlates of Psychological Distress (K-10)

Unadjusted and adjusted models for the odds of reporting higher median K-10 total scores are shown in Table 4. After controlling for the effects of salient predictors from the unadjusted model, the adjusted model indicated that satisfaction with life balance, satisfaction with social support and (older) age were associated with lower (i.e., better) psychological distress scores.

#### Correlates of Alcohol Consumption (AUDIT-C)

In the unadjusted model, the only significant correlate for alcohol consumption (AUDIT-C) scores was satisfaction with life balance (*p* = 0.001), where satisfaction was associated with lower reported alcohol consumption. Given that satisfaction with life balance was the only significant correlate in the unadjusted model, no multivariate modeling was performed for this outcome variable.

#### Correlates of Sleep Disturbance (ASSQ)

Unadjusted and adjusted models of sleep disturbance are shown in Table 5. In the adjusted model, the odds of reporting higher ASSQ scores (indicative of elevated sleep disturbance) was only significantly associated with satisfaction with social support.

## Discussion

To our knowledge, this is the first study to examine the rates of common mental health concerns in HPSS, and to compare coach and HPSS mental health and well-being outcomes to published elite athlete data. The results show that coaches and HPSS report similar mental health symptom profiles and that the prevalence of mental health and well-being concerns is largely consistent with those previously observed in elite athletes [3, 15, 27, 28]. Satisfaction with social support and satisfaction with life balance were found to be key correlates of mental health and well-being outcomes. These results suggest that coaches and HPSS are susceptible to the pressures associated with high-performance sports settings and may benefit from access to appropriate mental health supports and strengthening the known protective factors for mental health that are increasingly being advocated for elite athletes (e.g., [31]).

### Comparing Estimated Rates of Mental Health Concerns with Published Athlete Data

The proportion of coaches and HPSS reporting mental health symptoms at a level that would warrant professional treatment (i.e., caseness) was approximately 40%. This rate is similar to that previously observed in a comparable elite athlete sample (35%) [3], but notably higher than community norms (see Additional file 1: Table S3). The proportion of coaches meeting caseness criteria (43.6%) was consistent with the rate of 39.5%, which was reported in a recent study of coaches by Kegelaers and colleagues [14], as was the proportion of HPSS meeting caseness criteria (40.1%). Notably, 34.5% of participants also reported accessing psychological treatment at some stage, suggesting responsiveness to mental health symptoms and openness to treatment among this group. The relatively high proportion of coaches and HPSS in this sample meeting caseness criteria (and reporting past help-seeking) suggests that it is not just competing (as an athlete) in elite sports that confers a risk for mental ill health, but operating within the broader social ecology of high-performance sport and the attendant pressures associated with these systems that may be relevant.

In further support of hypotheses 1 and 2, no difference was observed between psychological distress reported by coaches or HPSS compared to athletes. Coach and HPSS psychological distress was also similar to community norms (Additional file 1: Table S3) [32, 33]. While these findings do not align with Kim and colleagues’ study [13], which found the prevalence of reported depressive symptoms among coaches to be similar to the general community, but lower than elite athletes, they are consistent with Kegelaers and colleagues’ results, which found similar reporting of mental health symptoms between elite-level coaches and athletes [14]. These mixed findings likely reflect the early stage of empirical research into elite coach mental health and indicate the need for further attention. Possible differences across studies are also important to consider in relation to sport-specific factors, such as period of the competitive season, performance outcomes or sport type. The lack of differences in mental health symptoms observed between HPSS and elite athletes in this study is a unique contribution to the literature and supports the need for investment in mental health support services that are offered to all key stakeholders in elite sports settings rather than only athletes (such as the AIS Mental Health Referral Network [34], which has been expanded to support coaches and HPSS, in addition to current and former athletes).

Risky alcohol consumption was reported by a significantly higher proportion of coaches and HPSS than elite athletes [15], with 48.1% of coaches and 39.0% of HPSS reporting potentially risky levels of alcohol consumption (with no significant difference between coaches and HPSS). The reporting of elevated alcohol consumption in the coach and HPSS groups relative to athletes may relate to coaches and HPSS not being subject to the same physical fitness and performance demands as elite athletes (including physical metrics such as skin fold tests and weigh-ins), who consistently report low rates of alcohol and other substance use [35, 36]. However, coaches and HPSS reported significantly *lower* levels of alcohol consumption than community norms (Additional file 1: Table S3) [37]. To our knowledge, no other studies have examined alcohol consumption in HPSS and only two other studies [14], 15 have examined alcohol consumption in elite coaches, reporting lower levels than those observed here. Future studies could consider coach and HPSS alcohol consumption in response to performance outcomes (e.g., poor performance) and work-related stress in order to ascertain optimal methods to support such staff in high-pressure and high-performance (including ‘win at all costs’) settings.

The responses of the HPSS group on the GHQ-28 indicated higher reporting of anxiety/insomnia and social dysfunction symptoms, and lower severe depression symptoms, than the comparable elite athlete sample. It is unclear why these differences were found for HPSS and not coaches. One possible reason regarding the anxiety symptoms is that the HPSS sample represented a more even gender distribution than the coach sample (which had a higher proportion of male participants). Given that previous research has shown elevated anxiety in females relative to males among elite athlete samples [10] and in the general population [38], the elevated scores on the anxiety/insomnia and social dysfunction subscales in the HPSS but not coach group are perhaps unsurprising (given this difference in gender breakdown between groups).

### Similarities and Differences Between Coaches and HPSS

Coaches and HPSS reported similar mental health and well-being scores on all major outcome measures. This is consistent with the finding that an almost identical rate of coaches and HPSS reported seeking treatment for a mental health problem (approximately 1 in 3 for both).

In further support of H2, coaches and HPSS also reported similar adverse life events and similar strategies for managing stress and mental well-being. Of note, approximately half of the coaches and HPSS reported feeling undervalued or underpaid (across the lifetime). Sporting organizations are encouraged to consider strategies for highlighting the value of members of the daily training environment, whether through financial compensation, public recognition, enhancing the organizational culture or other strategies. Research using qualitative methods would be particularly valuable for exploring ways that coaches and HPSS can be supported to feel better valued.

Additionally, approximately half of the coaches and HPSS reported satisfaction with life balance and satisfaction with their available social support. This is consistent with previous work, where elite coaches also reported having a lack of life balance (e.g., lack of time to spend with family, competing responsibilities) [13]. Given that both satisfaction with life balance and satisfaction with social support were key protective factors for mental ill health among elite coaches and HPSS, there should be dedicated efforts to strengthen these factors, particularly during periods of higher workload and pressure (e.g., in the lead-up to major competition).

### Correlates of Mental Health and Well-Being

The most robust correlates in this sample were satisfaction with social support and satisfaction with life balance, which both showed negative associations with symptoms of common mental health concerns and psychological distress. Satisfaction with social support was also negatively associated with sleep disturbance, while satisfaction with life balance was negatively associated with alcohol consumption. Social support and life balance are well-known modifiable protective factors for mental health [39, 40]. Enhancing these protective factors in coaches and HPSS not only has the potential to directly enhance coach and HPSS well-being, but also has the potential to improve these factors among athletes through the role modeling of a healthy life balance and the importance of maintaining supportive relationships.

Few sport-related or demographic variables were significantly associated with the outcome measures in the adjusted analyses, with the exception that younger age was associated with elevated psychological distress. No significant gender differences or differences according to sport type were found in the current sample, which supports findings from prior studies, which also report a lack of gender differences in mental health symptoms among elite coaches [13, 14]. However, this is in contrast to previous research with elite athlete samples, which show elevated reporting of mental health symptoms in females [10] and athletes from individual sports [11]. Taken together, these findings tentatively suggest the potential for a differential effect of gender between athletes versus non-athlete members of the daily training environment in terms of mental health and well-being outcomes. Future studies would benefit from including a greater number of female participants to further examine possible gender differences in mental health and well-being outcomes in coach and HPSS samples.

### Limitations

This study has several limitations, chief among them being the response rate of 31.5%. It is possible (though unable to be determined) that coaches and HPSS with experience of mental health symptoms were more inclined to participate in the survey, leading to a non-representative and potentially biased sample. Furthermore, the use of cross-sectional data does not allow for consideration of changes in mental health and well-being outcomes across key time points. In particular, it would be valuable to investigate possible changes in reported life balance, mental health symptoms, alcohol consumption and sleep disturbance across the competitive season and following various performance outcomes. Investigating the relationship between performance and mental health outcomes is paramount, particularly in the context of findings by Kegelaers et al. [14], who reported that performance-based stressors had the highest self-reported impact on Dutch and Flemish coach mental health (followed by organizational stressors, then personal stressors). It is also recommended that future studies consider the examination of the broader *spectrum* of mental health experiences, rather than merely presence of mental health symptomatology. Finally, the survey was launched in March 2020 and closed in May 2020 (by which time the postponement of the Tokyo Olympics and Paralympics had been confirmed). The significant disruption to participants in light of the pandemic and attendant lockdowns may have influenced the survey response rate, although no major differences in the main outcomes according to reported concern about the pandemic were found.

## Conclusions

This paper describes the mental health and well-being of 252 elite coaches and HPSS from a range of sports within Australia’s high-performance sport system. Results showed similar profiles between coaches and HPSS on the included measures. Despite relatively high rates of probable caseness, coaches and HPSS scored favorably on the measure of alcohol consumption (in comparison with community data) and a relatively high proportion reported previous help-seeking for mental health problems. Coaches and HPSS also reported similar mental health and well-being profiles as elite athlete samples, with the exception of higher alcohol consumption in coaches and HPSS relative to athletes. Some mixed findings were observed with the HPSS group, where they reported elevated symptoms of anxiety/insomnia and social dysfunction, but lower scores on the measure of severe depression, relative to athletes. Although only approximately half of the coaches and HPSS reported satisfaction with their life balance and their social support, these were the most robust correlates of the mental health and well-being outcomes included in this paper, where both acted as key protective factors for mental ill health. Based on these findings, sporting organizations should be aware that members of the daily training environment are relatively likely to experience mental health difficulties and should aim to strengthen key protective factors in members of the daily training environment, including optimal life balance and social supports.

## Supplementary Information

**Additional file 1.** **Supplementary Table 1.** Adverse events reported by coaches and HPSS. **Supplementary Table 2.** Coach and HPSS reported strategies for managing stress and mental wellbeing. **Supplementary Table 3.** Reported mental health symptoms among coaches and HPSS compared to published community samples.

## Data Availability

The data that support the findings of this study are available on request from the corresponding author [VP] and with the permission of the Australian Institute of Sport. The data are not publicly available due to them containing information that could compromise research participant privacy.

## Abbreviations

HPSS: High-performance support staff
AIS: Australian Institute of Sport
GHQ-28: General Health Questionnaire-28 items
K-10: Kessler Psychological Distress Scale-10 items
ASSQ: Athlete Sleep Screening Questionnaire
AUDIT-C: Alcohol Use Disorders Identification Test-Consumption
