# Supplementary material for: Prevalence and Correlates of Mental Health Symptoms and Well-Being Among Elite Sport Coaches and High-Performance Support Staff
Source: Sports Med Open. 2022 Jul 6;8:89. doi: 10.1186/s40798-022-00479-y (PMC9259770; doi:10.1186/s40798-022-00479-y)
Supplement: Supplementary file 1 — Additional file 1. Supplementary Table 1. Adverse events reported by coaches and HPSS. Supplementary Table 2. Coach and HPSS reported strategies for managing stress and mental wellbeing. Supplementary Table 3. Reported mental health symptoms among coaches and HPSS compared to published community samples. [file 40798_2022_479_MOESM1_ESM.docx]

**Prevalence and correlates of mental health symptoms and wellbeing amongst elite sport coaches and high performance support staff**

Vita Pilkington^1,2^, Simon Rice^1,2^, Courtney C. Walton^1,2^, Kate Gwyther^1,2^, Lisa Olive^1,2,3^, Matt Butterworth^4^, Matti Clements^4^, Gemma Cross^4^, Rosemary Purcell^1,2^

^1^ The Centre for Youth Mental Health, The University of Melbourne, Melbourne, Australia

^2^ Elite Sports and Mental Health, Orygen, 35 Poplar Road, Parkville, VIC 3052, Australia

^3^ School of Psychology, Faculty of Health, Deakin University, Geelong, VIC 3220, Australia

^4^ Australian Institute of Sport, People Wellbeing and Engagement, Bruce, Australia

Corresponding author: Vita Pilkington, Centre for Youth Mental Health, The University of Melbourne, Melbourne VIC, Australia. Email: [vita.pilkington@unimelb.edu.au](mailto:vita.pilkington@unimelb.edu.au)

**Supplementary Information**

##### Supplementary Table 1 Adverse events reported by coaches and HPSS

| Adverse event |  | Coach  % (n) | HPSS  % (n) |
| --- | --- | --- | --- |
| I suffered from serious illness or injury | Past year  Lifetime | 11.1 (9)  33.3 (27) | 5.6 (10)  42.9 (76) |
| A close relative suffered from serious illness, injury or assault | Past year  Lifetime | 12.3 (10)  51.9 (42) | 11.3 (20)  49.7 (88) |
| A person close to me died | Past year  Lifetime | 8.6 (7)  54.3 (44) | 13.6 (24)  49.2 (87) |
| I separated from my partner/long-term relationship ended | Past year  Lifetime | 1.2 (-)  25.9 (21) | 2.3 (-)  35.0 (62) |
| I had a serious problem with a close friend, relative or neighbor | Past year  Lifetime | 1.2 (-)  14.8 (12) | 3.4 (6)  18.1 (32) |
| Experienced financial problem/hardship | Past year  Lifetime | 9.9 (8)  25.9 (21) | 4.0 (7)  26.0 (46) |
| I was stalked by a fan | Past year  Lifetime | 1.2 (-)  6.2 (5) | 0.0 (-)  3.4 (6) |
| I experienced discrimination | Past year  Lifetime | 2.5 (-)  23.5 (19) | 3.4 (6)  21.5 (38) |
| I was harassed or abused on social media | Past year  Lifetime | 2.5 (-)  17.3 (14) | 2.3 (-)  8.5 (15) |
| I was the victim of a crime | Past year  Lifetime | 1.2 (-)  19.8 (16) | 1.7 (-)  15.3 (27) |
| I felt under-valued, including under-paid | Past year  Lifetime | 8.6 (7)  54.3 (44) | 16.9 (30)  48.6 (86) |
| I had a problem with the police involving a court appearance | Past year  Lifetime | 3.7 (-)  3.7 (-) | 0.0 (-)  2.8 (5) |
| Experienced uncertainty around receiving direct financial support (dAIS) | Past year  Lifetime | 3.7 (-)  8.6 (7) | 0.6 (-)  1.7 (-) |

Note: dash (-) indicates n<5.

##### Supplementary Table 2 Coach and HPSS reported strategies for managing stress and mental wellbeing

| Managing mental wellbeing activity | Coach  % (n) | HPSS  % (n) |
| --- | --- | --- |
| Take time away from sport to relax | 44.2 (34) | 47.3 (79) |
| Meditate | 9.1 (7) | 18.0 (30) |
| Talk with a friend/partner | 71.4 (55) | 83.8 (140) |
| Exercise for pleasure | 67.5 (52) | 80.2 (134) |
| Use relaxation techniques | 23.4 (18) | 20.4 (34) |
| Practice gratitude | 23.4 (18) | 26.9 (45) |
| Plan important aspects of life | 37.7 (29) | 47.9 (80) |
| Make sure to eat well | 53.2 (41) | 61.1 (102) |
| Sleep | 61.0 (47) | 71.9 (120) |
| Try to problem solve issues/problems | 55.8 (43) | 53.9 (90) |
| Do things I’m good at to feel accomplished | 29.9 (23) | 25.7 (43) |
| Yoga/Pilates | 14.3 (11) | 22.2 (37) |
| Speak to a mental health professional | 11.7 (9) | 14.4 (24) |
| Practice mindfulness | 16.9 (13) | 25.7 (43) |
| Go away on holiday | 32.5 (25) | 44.9 (75) |
| Enjoy regular enjoyable activities (e.g. walking the dog, listening to music) | 59.7 (46) | 74.3 (124) |
| Nothing | 6.5 (5) | 3.6 (6) |
| I don’t know how to manage my mental wellbeing or stress | 3.9 (-) | 1.8 (-) |

##### Supplementary Table 3 Reported mental health symptoms among coaches and HPSS compared to published community samples

| Measure |  | Coaches | HPSS | Published community samples |
| --- | --- | --- | --- | --- |
| *GHQ-28* |  |  |  |  |
| Total score | **M (SD)** | 20.62 (9.87)** | 20.70 (9.95)** | 16.5 (9.8) [41] |
| Somatic complaints | **M (SD)** | 5.56 (3.42)** | 5.68 (3.76)** | 4.2 (3.6) [41] |
| Anxiety/insomnia | **M (SD)** | 6.27 (4.53)* | 6.38 (4.46)** | 5.0 (4.1) [41] |
| Social dysfunction | **M (SD)** | 7.58 (2.39)** | 7.82 (2.71)** | 6.1 (2.1) [41] |
| Severe depression | **M (SD)** | 1.27 (2.58) | 0.77 (1.53)* | 1.1 (2.5) [41] |
| Caseness | **% (n)** | 43.6** | 40.1** | 19.5 [42] |
| *K-10* |  |  |  |  |
| Total score | **M (SD)** | 15.58 (4.95) | 15.75 (5.25)* | 14.5 (-) [32] |
| High to very high distress | **%** | 10.3 | 15.5* | 9.6 [32] |
| *AUDIT-C* |  |  |  |  |
| Total | **M (SD)** | 3.88 (2.55)* | 3.18 (2.12)** | 4.70 (2.66) [36] |
| Risky alcohol consumption | **%** | 48.1 | 39.0** | 57.1 [36] |
| Satisfaction with life total score | **M (SD)** | 25.22 (5.75)* | 25.91 (5.40)** | 23.08 (6.12) [43] |

Note: Comparisons were performed between the coach sample vs published community samples and between the HPSS sample vs published community samples, **p*< .01, ***p*< .001. For the GHQ-28, the cut-off score used was 5 or more indicating probable caseness. For risky alcohol consumption, the cut-offs used were 4 or more for females and 5 or more for males. K-10 scores between 22-50 were suggestive of ‘high to very high’ distress. ASSQ scores have not been reported in this table as this measure was designed for athletes and therefore community comparisons were not available/appropriate.
